# To characterize contrast detection, noise should be extended, not localized

**DOI:** 10.3389/fpsyg.2014.00749

**Published:** 2014-07-11

**Authors:** Rémy Allard, Jocelyn Faubert

**Affiliations:** ^1^INSERM, U968, ParisFrance; ^2^Institut de la Vision, Sorbonne Universités – University Pierre and Marie Curie, UMR_S 968, ParisFrance; ^3^CNRS, UMR_7210, ParisFrance; ^4^Visual Psychophysics and Perception Laboratory, Université de Montréal, Montréal, QCCanada; ^5^NSERC-Essilor Industrial Research Chair, Montréal, QCCanada

**Keywords:** external noise paradigm, extended noise, 0D noise, contrast detection, contrast discrimination

## Abstract

Adding noise to a stimulus is useful to characterize visual processing. To avoid triggering a processing strategy shift between the processing in low and high noise, Allard and Cavanagh (2011) recommended using noise that is extended as a function of all dimensions such as space, time, frequency and orientation. Contrariwise, to avoid cross-channel suppression affecting contrast detection, Baker and Meese (2012) suggested using noise that is localized as a function of all dimensions, namely “0D noise,” which basically consists in randomly jittering the target contrast (and, for blank intervals or catch trials, jittering the contrast of an identical zero-contrast signal). Here we argue that contrast thresholds in extended noise are *not* contaminated by noise-induced cross-channel suppression because contrast gains affect signal and noise by the same proportion leaving the signal-to-noise ratio intact. We also review empirical findings showing that detecting a target in 0D noise involves a different processing strategy than detecting in absence of noise or in extended noise. Given that internal noise is extended as a function of all dimensions, we therefore recommend using external noise that is also extended as a function of all dimensions when assuming that the same processing strategy operates in low and high noise.

Noise can be used to characterize visual processing in noiseless conditions. For instance, contrast detection threshold in absence of noise is limited by both internal noise and the ability of detecting the signal embedded in noise, namely, calculation efficiency, which is inversely proportional to the smallest signal-to-(internal) noise ratio required to detect the signal. These two factors can be estimated by measuring contrast thresholds in low and high noise levels (Pelli, 1981; Pelli and Farell, 1999). In high noise, internal noise has negligible impact and the smallest signal-to-(external) noise ratio required to detect the signal (i.e., calculation efficiency in high noise) can be calculated given the contrast threshold in a given high external noise level. By assuming that the smallest signal-to-(internal) noise ratio required to detect the signal is the same as the measured smallest signal-to-(external) noise ratio required to detect the signal in high noise (i.e., assuming that the calculation efficiency in low noise is the same as the measured calculation efficiency in high noise), the relative impact of internal noise can be estimated, which is referred to as the internal equivalent noise. Thus, measuring contrast thresholds in low and high noise while assuming that the smallest signal-to-noise ratio required to detect the signal (i.e., calculation efficiency) is the same in low and high noise enables the measurement of factors limiting contrast thresholds in absence of noise, that is, internal equivalent noise and calculation efficiency.

Different types of noise can be used (**Figure 1**). Typically, noise turns on and off with the target (i.e., temporally localized) and appears at the target location (i.e., spatially localized) or over slightly larger area. As a function of orientation and frequency, noise is often extended (e.g., white noise), that is, it has a wide spectral energy spectrum across orientations and frequencies. Nonetheless, it is not unusual to filter the noise to keep only a range of frequencies and orientations (or even only one orientation as in **Figure 1**).

**FIGURE 1:**
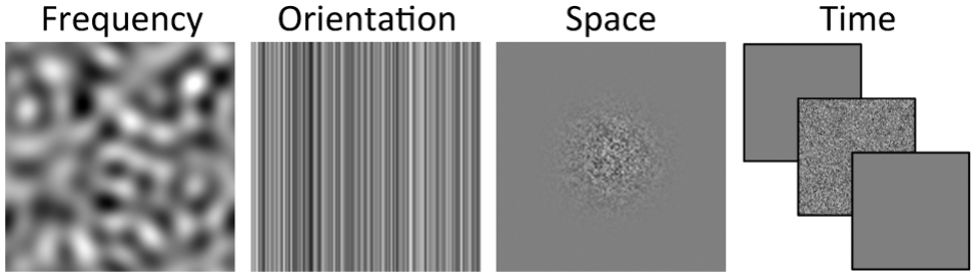
**Different types of noise that are localized relative to the frequency (e.g., contains only frequency around the frequency of the target), orientation (e.g., contains only the orientation of the target), space (e.g., occurs only at the target location) or time (e.g., turn on and off with the target).** The target is not illustrated.

Typically, experimenters arbitrarily select one type of noise that is localized relative to some dimensions and extended relative to others, and usually implicitly assume that the smallest signal-to-(internal) noise ratio required to detect the signal is the same as the measured signal-to-(external) noise ratio. This assumption enables the use of external noise to characterize processing in noise free displays. However, some recent studies suggest that this assumption can be violated when using some types of noise. Allard and Cavanagh (2011) argued that the detection strategy is not always noise-invariant, as the most sensitive processes in one noise type may not be the most sensitive processes in another noise type. For instance, in noise that is spatially localized (appears only at the target location) and temporally extended (i.e., continuously present), the best detection strategy could consist in detecting a temporal variation of response within a given channel, but in noise that is spatially extended and temporally localized, the best strategy could rather consist in detecting a spatial variation. Two distinct strategies will likely have distinct calculation efficiencies. Thus, to assume that calculation efficiency in absence of noise is the same as in high noise, the same processing strategy must operate in absence and presence of noise. To avoid different processing strategies operating in absence of noise and in high noise, external noise should match, as much as possible, the characteristics of internal noise (except for contrast). Internal noise is extended as a function of all dimensions as it occurs at all orientations and frequencies, it is not present only at the target location and it does not turn on and off with the target. Allard and Cavanagh (2011) therefore recommended using noise that is extended as a function of all dimensions, such as space, time, frequency and orientation (**Figure 2** right).

**FIGURE 2:**
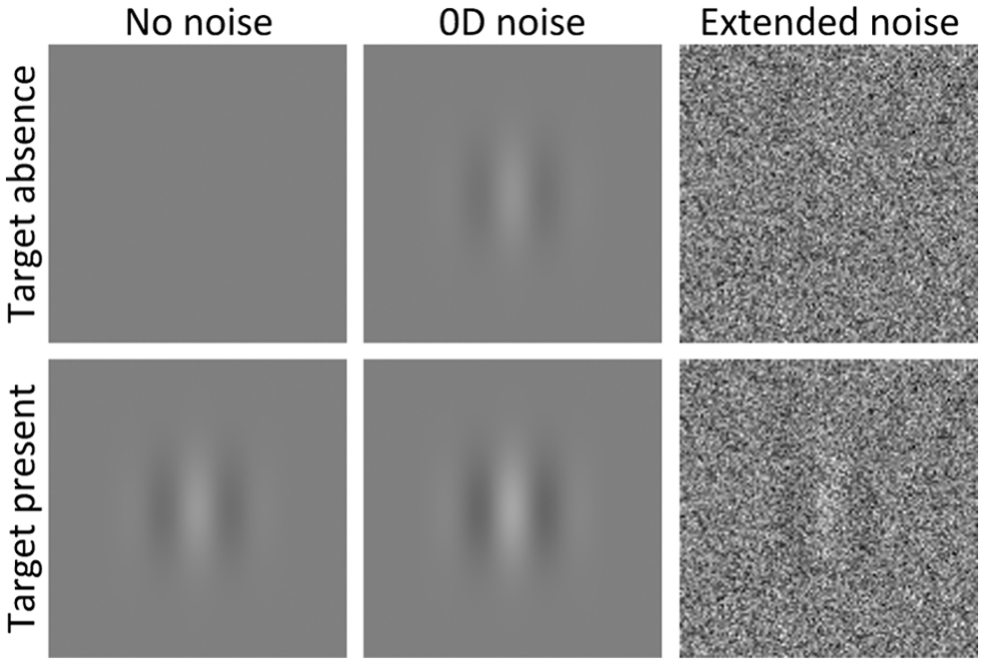
**Two different intervals (target absent, top, and present, bottom) in absence of noise (left), in noise that is localized as a function of all dimensions (i.e., 0D noise, center) and in noise that is extended as a function of all dimensions (right).** Note that 0D noise basically consists in randomly jittering the target contrast (and, when the target is absent, jittering the contrast of an identical zero-contrast signal). Negative contrasts correspond to a polarity reversal (not illustrated).

Contrariwise, Baker and Meese (2012) criticized the use of extended noise due to cross-channel suppression as the response within one channel tends to suppress the responses of other nearby channels. For instance, noise extended as a function of orientation will introduce noise not only in the channels tuned to the signal orientation, but also to channels tuned to all other orientations, which may suppress the response within the relevant channels. To avoid cross-channel suppression affecting contrast detection threshold in high noise, Baker and Meese (2012) suggested to use noise that is localized as a function of all dimensions, which they refer to as “0D noise” (**Figure 2** center), which basically consists in randomly jittering the target contrast (and, for blank intervals or catch trials, jittering the contrast of an identical zero-contrast signal).

In sum, many experimenters use noise that is localized as a function of some dimensions and extended as a function of others, and implicitly assume that the calculation efficiency in low noise is the same as the measured calculation efficiency in high noise. However, given that internal noise is extended, this assumption may be violated in localized noise if different processing strategies operate in localized and extended noise, which would result in different processing strategies in low localized noise (i.e., when internal extended noise dominates) and high localized noise. It may also be violated in extended noise if noise-induced cross-channel suppression affects the measurement of the calculation efficiency in high noise. The objective of the present study was to determine which noise type (localized or extended) should be used to avoid violating the assumption that the calculation efficiency in low noise is the same as the measured calculation efficiency in high noise, which is necessary to characterize detection processing in noiseless conditions (e.g., measure internal equivalent noise and calculation efficiency). Note that because we see no reasons a priori why the noise should be localized as a function of some dimensions and extended as a function of others, the current article focused on the two extreme cases: noise extended or localized as a function of all dimensions. On the one hand, if noise-induced cross-channel suppression affects the measured calculation efficiency, this will likely be the case for any dimension. On the other hand, if noise should be analogous to internal noise to avoid triggering a processing strategy shift, then it should be extended as a function of all dimensions. The present article first investigated if adding 0D noise (i.e., noise localized as a function of all dimensions) triggers a shift in processing strategy and then investigated if noise-induced cross-channel suppression affects contrast thresholds in extended noise.

## NOISE-INVARIANT PROCESSING ASSUMPTION

0D noise has the advantage that it cannot induce cross-channel suppression because it contains energy only within the relevant channels. Thus, the usefulness of 0D noise to characterize the *detection* process depends on whether the same processing strategy operates in 0D noise as in absence of noise. In a two-interval forced-choice paradigm (2IFC), a contrast detection task consists in one interval containing the signal at a given contrast level and the other interval is blank (or contains an identical zero-contrast signal). For such a detection task, adding 0D noise consists in adding an independent contrast jitter to both intervals. As a result, a signal is presented in both intervals (e.g., **Figure 2** center) and the task consists in discriminating the interval containing the highest contrast (while considering a contrast opposite to the signal as a negative contrast). In other words, a contrast detection task in 0D noise is processed as a contrast *discrimination* task (Allard and Faubert, 2013). Thus, if the processing strategies underlying contrast detection and discrimination tasks differ, then 0D noise could not be used to investigate the contrast detection process. Nevertheless, Baker and Meese (2013) argued that 0D noise can be used to characterize the *detection* process because, they claimed, a detection task is always processed as a discrimination task. In other words, they suggest that the processing strategy is the same for contrast detection and discrimination tasks. If the same processing strategy operates for contrast detection and discrimination tasks, then 0D noise could indeed be used to characterize the detection process. On the other hand, if contrast detection, and discrimination tasks involves distinct processing strategies, this would disqualify the use of 0D noise to characterize the detection process and would provide further evidence that different processing strategies can operate in the absence of noise and presence of localized noise. Here, we review empirical findings suggesting that the detection strategy in noiseless conditions is not based on a discrimination strategy, as is the case for a detection task in 0D noise.

**FIGURE 3:**
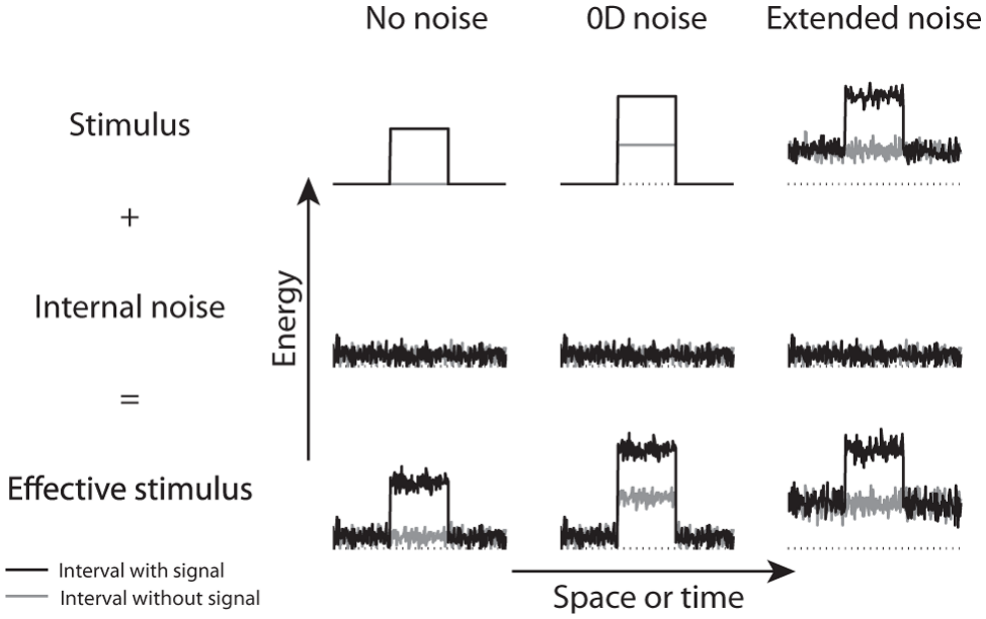
**Energy level when a target is present (black) or absent (gray) as a function of a given dimension (e.g., space or time) for three conditions: no noise (left column), 0D noise (or contrast discrimination, middle column) and extended noise (right column).** The top row represents the energy level of the external stimulus, the middle row represents internal noise added by the visual system and the bottom row represents the effective stimulus (i.e., the external stimulus summed with internal noise). The effective stimulus of the no and extended noise conditions have similar profiles, which is different from the one with the 0D noise that shows an important energy variation even in the absence of a signal. The dotted line represents the zero energy level. This figure was adapted from Allard et al. (2013).

The contrast discrimination processing strategy is straightforward: compare two responses from the two intervals and report the one with the highest. Although such a strategy could also be used for contrast detection, it is not necessarily the case. As we suggested elsewhere (Allard and Cavanagh, 2011; Allard et al., 2013), an alternative detection strategy consists in determining if a pattern can be distinguished from the noisy background (**Figure 3**). According to this processing strategy, detection thresholds do not depend on the ability to discriminate two responses (as soon as the target is detected in one interval, the task is trivial), but simply on the ability to distinguish a pattern from the noisy background. Conversely, discrimination thresholds do not depend on the ability to distinguish a pattern from the noisy background (this is usually trivial because both stimuli are generally suprathreshold), but on the ability to estimate and compare two contrasts. According to **Figure 3**, the discrimination strategy would consist in comparing the energy levels in the central portion of the two curves (which could operate in any noise condition), whereas the detection strategy could also consist in distinguishing a variation of energy relative to the background (which could not operate in 0D noise as the noise alone induces such a variation).

Allard and Cavanagh (2011) found empirical evidence that the detection strategy consists in distinguishing a pattern from the noisy background. They found that spatiotemporally localized noise (i.e., noise appearing only at the potential target spatiotemporal locations), which introduces energy easily distinguishable from the background (similar to the middle column in **Figure 3**), impaired the detection process and triggered a change in processing strategy: the processing strategy shifted from a detection strategy immune to crowding to a discrimination or recognition strategy that is sensitive to crowding. This processing strategy shift must be due to the spatiotemporal window of the noise and not to the noise *per se* because extended noise (i.e., background dynamic, white noise that is full screen and continuously displayed, **Figure 3** right column) was not found to affect the detection strategy. If the detection strategy in absence of noise consists in discriminating two activity levels, then there is no reason why this strategy would change in localized, but not extended noise. Allard and Cavanagh (2011) therefore suggested that the detection strategy in noiseless displays consists in distinguishing a pattern from the background internal noise, not comparing activity levels (which would be the same in localized and extended noise).

A particularity of the detection process is that it can be facilitated by the superposition of a low-contrast pedestal. Indeed, contrast discrimination functions (i.e., contrast discrimination thresholds as a function of the pedestal contrast), which show a gradual shift from a detection task (zero contrast pedestal) to a contrast discrimination task (high contrast pedestal), typically show a dip when plotted in contrast units: low contrast pedestals facilitate contrast detection thresholds and high contrast pedestals impair contrast discrimination thresholds (for a review, see Solomon, 2009). Such a dipper function was observed in absence of noise and in extended noise (Pelli, 1981) and led Pelli to state that “The dip is of great theoretical interest because it indicates that the process of detection is similar with and without the noise mask.” (p. 123). Indeed, similar patterns of results with and without noise suggest common underlying processes. If the detection strategy in 0D noise were the same as in absence of noise, then we would also expect a similar dip with 0D noise. However, this is obviously not the case because the detection thresholds in 0D noise are close to the ideal performance (Allard and Faubert, 2013; Baker and Meese, 2013) so substantial facilitation is impossible. This absence of facilitation in 0D noise, and the facilitation in noiseless and extended noise suggest that the detection strategy in 0D noise (i.e., contrast discrimination strategy) differs with the detection strategy in noiseless or extended noise conditions.

To add further evidence that the detection strategy does not consist in discriminating contrasts, but rather consists in distinguishing a pattern from the noisy background, we conducted an additional experiment. We compared contrast thresholds obtained using two 2IFC procedures. In the detection condition, one interval contained the target and the other was blank. In the phase-discrimination condition, one interval contained the target and the other contained the same target but with a reversed contrast polarity (i.e., negative contrast). Thus, for a given target contrast, the signed contrast difference between the two intervals in the phase-discrimination condition would be twice the one in the detection condition. If the processing strategy consists in comparing the signed contrast difference between the two intervals, then the contrast thresholds should be two times lower in the phase-discrimination condition. Indeed, the contrast difference between the two intervals would be the same in the two conditions when the contrast in the phase-discrimination condition would be half the contrast in the detection condition. Thus, we would expect the threshold in the detection condition to be twice the one in the phase-discrimination condition. Note that nonlinearities within the visual system could make this factor differ from 2. For instance, if the threshold depends on the energy difference (which is proportional to the squared contrast) between the two intervals (Raab et al., 1963) rather than the contrast difference, then we would expect the threshold in the detection condition to be $\sqrt{2}$ times higher than in the phase-discrimination condition (energy doubles when increasing contrast by a factor of $\sqrt{2}$). In any case, contrast thresholds would be non-negligibly lower in the phase-discrimination condition because, for a given target contrast, contrast difference (or energy difference) between the two intervals in the phase-discrimination condition would be twice the one in the detection condition. On the other hand, if the processing strategy consists in distinguishing a pattern from the noisy background, then the advantage in the phase-discrimination condition would only be due to the fact that two targets are presented compared to only one in the detection condition. The observer would have two chances instead of one to detect a target, so the observer would require a lower contrast level to obtain the same performance level. However, given that human observers have a sharp psychometric functions, performance drops rapidly when decreasing the target contrast so this advantage would only be of a factor of about 1.2 (Legge, 1984). Furthermore, this factor could be even less if the phase was not always discriminable when the target is detected because this would be a disadvantage in the phase-discrimination condition, but not in the detection condition.

### METHOD

The target to detect was a vertically oriented Gabor with a spatial frequency of 0.7 cycles/degree and a standard deviation of the Gaussian window of 0.5°. The 0D noise contrast was 0.06 (standard deviation of the Gaussian distribution). The extended noise was binary with elements of 2 × 2 pixels, resampled at 60 Hz and had a contrast of 0.32. The presentation time of each interval was 200 ms and the ISI was 500 ms. The contrast of the target was controlled by a 3-down-1-up staircase procedure (Levitt, 1971), which was interrupted after 12 inversions. To improve the luminance intensity resolution the Noisy-Bit method (Allard and Faubert, 2008) was implemented with the error of the green color gun inversely correlated with the error of the two other color guns, which made the 8-bit display perceptually equivalent to an analog display having a continuous luminance resolution. There were six block conditions (two tasks and three noise conditions, i.e., no noise, 0D noise and extended noise) that were performed three times each in a pseudorandom order. Contrast thresholds were estimated as the geometric mean of the last eight inversions of the three blocks. Two naïve and one of the authors participated to the experiment.

### RESULTS AND DISCUSSION

**FIGURE 4:**
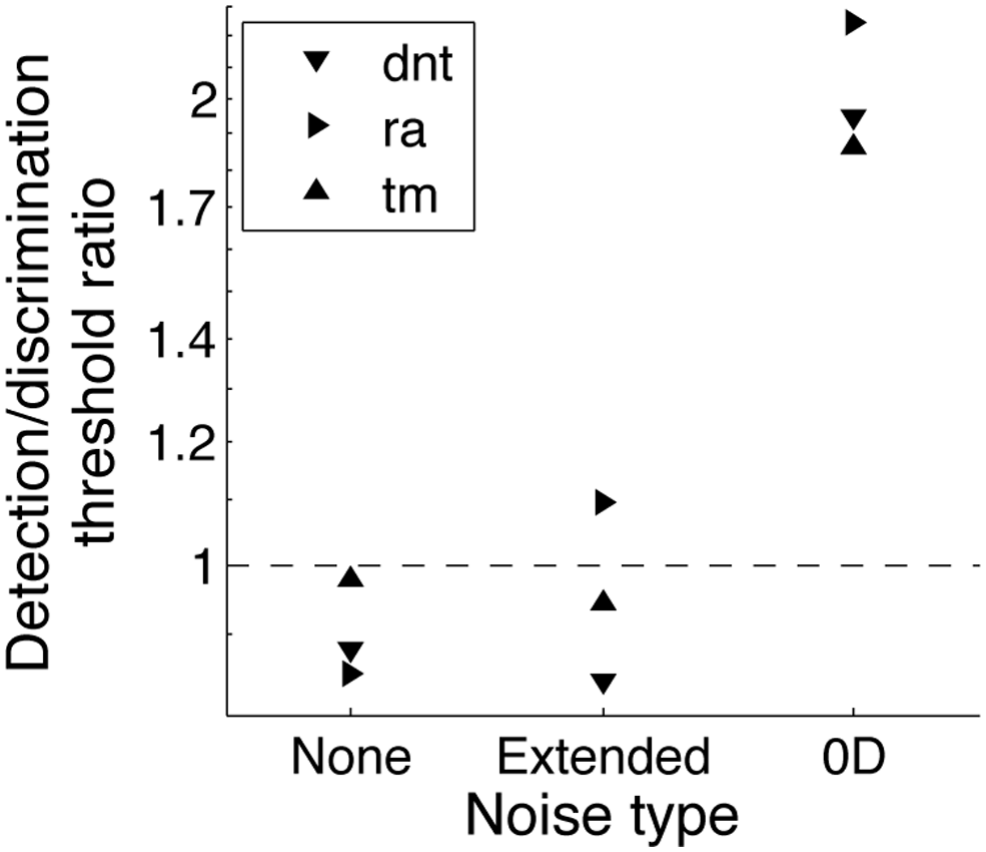
**Contrast thresholds for a 2IFC detection task relative to contrast thresholds for a 2IFC phase-discrimination task for three observers in three noise conditions: no noise, extended noise, and 0D noise**.

In 0D noise (noise independently added in the two intervals), presenting a negative target instead of a blank interval improved threshold performance by a factor of about 2 (**Figure 4**). This was expected given that the detection strategy in 0D noise is a contrast discrimination strategy so contrast threshold depends on the contrast difference between the two intervals. In absence of noise and in extended noise, however, doubling the contrast difference between the two intervals (by switching from a detection to a phase-discrimination condition) did not result in a substantial threshold increase as the threshold ratio between these two conditions was close to 1. This suggests that the detection strategy in these conditions does not consist in discriminating contrasts between the two intervals while considering contrasts opposite to the target as negative contrasts, but rather in distinguishing a pattern from the noisy background.

In sum, the patterns of results observed for a contrast detection task in absence of noise were similar to the ones in extended noise and drastically different in 0D noise. Adding a low contrast pedestal substantially improves contrast thresholds in absence of noise and in extended noise, but not in 0D noise. Conversely, replacing the blank interval with a negative target substantially improved contrast thresholds in 0D noise, but not in absence of noise or in extended noise. This double dissociation between detection tasks in extended noise (whether internal or external) and in 0D noise suggests that they involve different processing strategies. Contrast detection thresholds in absence of noise or in extended noise reflect the ability to distinguish a pattern from the noisy background, not to discriminate contrasts in different intervals as in 0D noise.

## MODERATE 0D NOISE LEVEL

The section above suggests that measuring contrast detection thresholds in *high* 0D noise (i.e., when the impact of internal noise is negligible) cannot be used to characterize the detection process because such a stimulus is processed by a discrimination strategy that is distinct from the detection strategy operating in absence of noise. On the other hand, *low* 0D noise is also not useful to characterize the detection process has it has a negligible impact. Nonetheless, this does not rule out the possibility that moderate levels of 0D noise could be used to characterize the *detection* process. The present section will investigate if moderate levels of 0D noise can be used to characterize the detection process.

**FIGURE 5:**
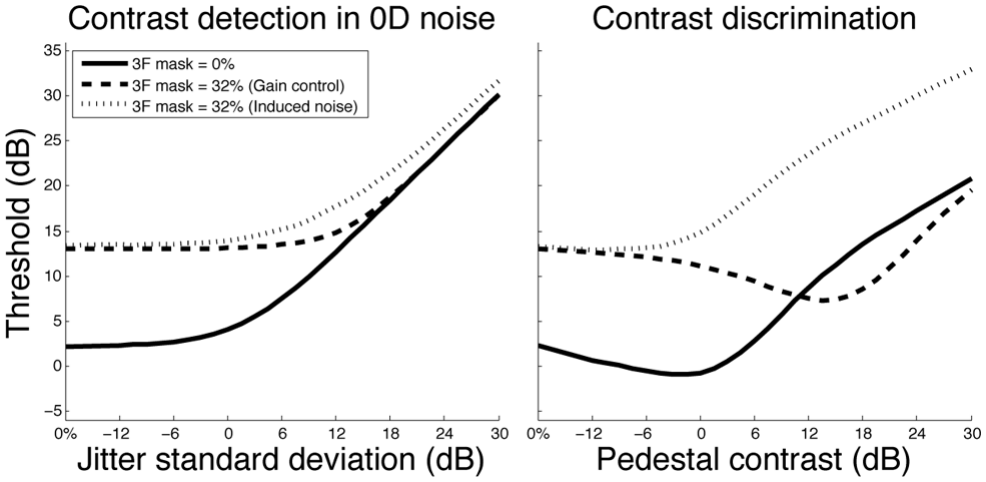
**Model predictions for contrast detection in 0D noise (left) and contrast discrimination (right) in absence of cross-channel masking (solid line), cross-channel masking due to gain control (dashed line) and cross-channel masking due to induced noise (dotted line).** For additional details on the simulations, see Baker and Meese (2013).

To empirically demonstrate the usefulness of 0D noise to characterize the detection process, Baker and Meese (2013) conducted an experiment in which they measured contrast detection thresholds as a function of noise contrast. Such a function usually shows, on a log–log plot, a smooth transition from a flat asymptote to a rising asymptote with a slope of 1 (e.g., **Figure 5** left). The flat asymptote can be evaluated by measuring detection threshold in absence of noise (or low noise). The rising asymptote in 0D noise can be evaluated by measuring contrast threshold in high 0D noise, but is known a priori as the task is trivial and the performance corresponds to the performance of an ideal observer (Allard and Faubert, 2013; Baker and Meese, 2013). Even though both asymptotes can be known without measuring any threshold in 0D noise, Baker and Meese (2013) showed that measuring contrast detection threshold as a function of 0D noise can be useful because different models predict different transitions between these two asymptotes. For instance, the gain control model would predict a smoother transition between the two asymptotes than the noise induce model (**Figure 5** left, see Baker and Meese, 2013, for model details). Given that a detection task is based on a detection strategy in low noise and a discrimination strategy in high 0D noise, the question is then to determine whether characterizing the transition between the two asymptotes reveals properties of the detection or discrimination process.

Since 0D noise in a 2IFC procedure consists in contrast jittering both intervals independently, many trials in 0D noise are useless (even near threshold) because they can easily be discriminated, especially at high 0D noise contrasts. This leaves few trials in which the two stimuli have similar contrasts and the response is not trivial and will thereby depend on human factors, such as the ability to discriminate contrasts. Thus, if the 0D noise contrast is high enough to affect detection threshold, but not too high so that there is a non negligible proportion of trials in which both contrasts cannot be discriminated (i.e., around the transition between the two asymptotes), then contrast detection threshold in 0D noise would depend on contrast discrimination threshold. So if different models predict different contrast discrimination thresholds, they would also predict different contrast detection thresholds in moderate 0D noise. In other words, contrast detection threshold in moderate 0D noise would be an indirect, noisy measure of the contrast discrimination threshold. To illustrate this, we have replicated Baker and Meese’s (2013) simulations for contrast detection threshold as function of 0D noise (**Figure 5** left) and ran the exact same simulations for a contrast discrimination task (i.e., the 0D noise was replaced by a pedestal, **Figure 5** right). Specifically, contrast thresholds as a function of external noise contrast (**Figure 5** left) and pedestal contrast (**Figure 5** right) were estimated by simulating trials using a standard detection model in which there was no masking (solid lines), the standard gain control model in which cross-channel masking is induced by suppression (dashed lines) and the noise-induced model in which cross-channel masking is induced by increasing internal noise (dotted lines). As illustrated in **Figure 5**, the two masking models, which affect contrast detection thresholds in absence of noise by the same proportion, predicted different contrast *discrimination* thresholds. This substantial contrast discrimination threshold difference directly explains the small contrast detection threshold difference in 0D noise. This shows that contrast detection threshold in moderate 0D noise is an indirect measure of the contrast discrimination process and that this experiment addresses the properties of the discrimination process, not the detection process.

Baker and Meese (2013) also showed that the two models predict different double pass consistencies. However, this property also directly depends on contrast discrimination thresholds. Indeed, the model predicting the higher contrast discrimination threshold will have the higher double pass consistency, as there will be more trials in which the two contrasts are discriminated. Given that the shape of the transition between the two asymptotes is directly related to contrast discrimination thresholds, we conclude that 0D noise could be used to investigate processing properties of the *discrimination* process, not detection process. In most cases, however, it would probably be more efficient to directly measure contrast discrimination thresholds. Nonetheless, even if there were some conditions in which measuring “detection” thresholds in 0D noise could be particularly useful to characterize the *discrimination* process, this would still not imply that measuring contrast detection thresholds in 0D noise can be useful to characterize the *detection* process.

## NOISE-INDUCED CROSS-CHANNEL SUPPRESSION

Baker and Meese (2012, 2013) argued that the use of white noise, which is extended as a function of frequency and orientation, is not suitable to measure internal equivalent noise because it induces cross-channel suppression affecting the measurement of contrast detection threshold in high noise thereby contaminating the measurement of the calculation efficiency. If the measurement of calculation efficiency in high noise were affected by noise-induced cross-channel suppression, then the assumption that the calculation efficiency in low noise is the same as the measured calculation efficiency in high noise would be compromised. Since contrast detection threshold in low noise depends on the internal equivalent noise and the calculation efficiency in low noise, not knowing the calculation efficiency in low noise would also compromise the estimation of the internal equivalent noise. The objective of the present section was to investigate if the assumption that the calculation efficiency in low noise is the same as the calculation efficiency in high noise is invalidated in extended noise due to noise-induced cross-channel suppression. Fortunately, we find that noise-induced cross-channel suppression does not affect contrast detection thresholds in high, extended noise for several reasons.

First, cross-channel suppression due to white noise seems weak. The strength of cross-channel suppression can be measured by asking the observer to match the contrast of a noise-free stimulus with the contrast of the same stimulus embedded in noise. Baker and Meese (2012) conducted such an experiment with 2D localized noise and their results were noisy: in some conditions, the noise had almost no impact on the perceived contrast and in others it affected threshold by a factor of about 2. This noise-induced suppression was not sufficient to explain the entire noise-induced threshold elevation of a factor of about 4. These results are inconsistent with previous findings showing that spatiotemporally extended white noise had no effect on perceived contrast (Pelli, 1981). To clarify this, we conducted our own contrast matching experiment and found that extended noise had no effect on perceived contrast (data not shown), which is consistent with Pelli’s findings. Thus, determining if white noise affects perceived contrast (which would suggest some cross-channel suppression) remains an open question, but if it does, the effect would remain modest suggesting that noise-induced cross-channel suppression is weak at best.

Anyhow, determining if there is no or a weak noise-induced cross-channel suppression is irrelevant when measuring contrast thresholds in high noise. Any contrast gain affecting both the signal and the dominating noise source would have no impact on the signal-to-noise ratio and thereby would not affect contrast threshold. This is nicely illustrated by Baker and Meese’s (2013) gain control model in which cross-channel suppression would affect contrast thresholds in low noise, but not in high noise (**Figure 5**, left). Indeed, when internal noise dominates (i.e., in low noise), a contrast gain occurring before the internal noise would affect the signal but not the dominating noise source and would therefore affect the signal-to-noise ratio. In high noise, however, the contrast gain would affect both the signal and the dominating noise source leaving the signal-to-noise ratio intact. Thus, even if noise reduced the effective contrast within the relevant channel due to cross-channel suppression, this contrast reduction would not affect contrast thresholds.

Further evidence that noise-induced cross-channel suppression does not affect contrast thresholds in high noise comes from the fact that contrast thresholds in high noise are proportional to noise contrast (slope of 1 in log–log units as in **Figure 5**, left). This was first observed by Pelli (1981) and has been consistently replicated across many studies. To our knowledge, this fact has never been contradicted. This proportional relation between contrast threshold and noise contrast implies that contrast thresholds at distinct high noise contrasts result in the same signal-to-noise ratio and thereby the same measured calculation efficiency. The fact that the measured calculation efficiency in high noise is independent of the noise contrast even though extended noise induces more cross-channel suppression as its contrast is increased suggests that the measurement of the calculation efficiency is not affected by noise-induced cross-channel suppression. More generally, given that the signal-to-noise ratio required to detect the signal is independent of the noise contrast, there is no reason why this ratio would differ when the limiting noise source is internal only because the noise contrast is lower. We therefore conclude that noise-induced cross-channel suppression does not affect contrast thresholds in high noise and thereby does not compromise the assumption that the measured calculation efficiency in high noise is the same as the calculation efficiency in low noise and does not contaminate the measurement of calculation efficiency and internal equivalent noise limiting detection threshold in the absence of noise.

## CONCLUSION

Empirical findings suggest that different processing strategies operate for contrast detection in 0D noise compared to contrast detection in absence of noise and in extended noise. In 0D noise, the processing strategy consists in discriminating two contrasts, whereas in absence of noise (i.e., extended internal noise) and extended noise, the processing strategy consists in distinguishing a pattern from the noisy background. This suggests that different processing strategies operate in absence of noise and in 0D noise, which compromises the use of 0D noise to characterize the detection process operating in absence of noise (e.g., measure internal equivalent noise). Conversely, we found no evidence that the processing strategy differed in absence of noise and in extended noise, which suggests that extended noise could be used to characterize the detection process.

Baker and Meese (2012) suggested that high extended noise induces cross-channel suppression affecting contrast thresholds and thereby the measured calculation efficiency, which therefore could not be assumed to be the same as the calculation efficiency in absence of noise. However, this contrast reduction (if any) would not affect the contrast threshold as it would also affect the noise contrast thereby leaving intact the signal-to-noise ratio. This suggests that extended noise can be successfully used to characterize the detection process and measure internal equivalent noise.

In sum, the current study concludes that noise *extended* as a function of all dimensions can be used to characterize the contrast detection process, but noise *localized* as a function of all dimensions cannot. Nevertheless, many experimenters use noise that is localized as a function of some dimensions and extended as a function of others. In principal, any property difference between internal and external noise could result in different detection strategies in low and high noise. On the other hand, a property difference between internal and external noise does not necessarily imply different detection strategies. For instance, a processing strategy could rely only on the central portion of a large stimulus and would therefore be independent of whether there is noise outside the stimulus region or not (i.e., spatially extended or localized noise, respectively; e.g., Allard and Faubert, 2014). Similarly, the processing strategy of a stimulus presented for a long duration would likely be independent of whether there is noise before and after the stimulus presentation or not (i.e., temporally extended or localized noise, respectively). Nonetheless, the detection strategy of a briefly presented, large stimulus could depend on whether the noise is temporally localized or extended (e.g., Allard and Faubert, 2014) and the detection strategy of a small stimulus presented for a long duration would likely depend on whether the noise is spatially localized or extended. Thus, using noise that is localized as a function of some dimensions raises doubts that the same detection strategy operates in low and high noise and thereby questions the assumption that the calculation efficiency in absence of noise is the same as the measured calculation efficiency in high noise. Given that internal noise is extended as a function of all dimensions, we therefore recommend using external noise that is also extended as a function of all dimensions when assuming that the same processing strategy operates in low and high noise.

## Conflict of Interest Statement

The authors declare that the research was conducted in the absence of any commercial or financial relationships that could be construed as a potential conflict of interest.
